# Radiation Segmentectomy for Hepatocellular Carcinoma

**DOI:** 10.3390/curroncol31020045

**Published:** 2024-01-23

**Authors:** Muhamad Serhal, Farnaz Dadrass, Edward Kim, Robert J. Lewandowski

**Affiliations:** 1 Department of Radiology, Northwestern University Feinberg School of Medicine, Chicago, IL 60611, USA; muhamad.serhal@northwestern.edu; 2 Department of Radiology, Mount Sinai Health System, New York, NY 10029, USA; farnaz.dadrass@mountsinai.org (F.D.); edward.kim@mountsinai.org (E.K.)

**Keywords:** radioembolization, radiation segmentectomy, hepatocellular carcinoma, Barcelona Clinic Liver Cancer paradigm

## Abstract

The application of trans-arterial radioembolization (TARE) with Yttrium-90, historically a palliative treatment option for patients with advanced hepatocellular carcinoma (HCC), is evolving. Radiation segmentectomy (RADSEG), the segmental delivery of an ablative radiation dose, is a treatment option for patients with earlier-stage HCC. This review presents an in-depth exploration of RADSEG, emphasizing its technical considerations, dosimetry advancements, and patient selection. The integration of RADSEG into the Barcelona Clinic Liver Cancer (BCLC) paradigm will be highlighted. RADSEG outcomes concerning safety and efficacy will be explored and compared with traditional locoregional cancer treatments like trans-arterial chemoembolization (TACE), percutaneous thermal ablation, and surgical resection, with an eye on future directions and considerations.

## 1. Introduction

Liver cancer is the sixth most frequently diagnosed malignancy and stands as the fourth cause of cancer-related deaths globally. Hepatocellular carcinoma (HCC) accounts for approximately 80% of all liver cancer diagnoses [1]. HCC detection at an early cancer stage provides an opportunity for effective locoregional treatments. The application of trans-arterial radioembolization (TARE) with Yttrium-90 (Y90), historically a palliative treatment option for patients with advanced HCC, is evolving in this space. Spearheading this evolution is radiation segmentectomy (RADSEG), a technique characterized by the segmental delivery of an ablative radiation dose exceeding a threshold radiation absorbed dose, which offers a locoregional treatment option for patients diagnosed with earlier-stage HCC. This article provides an in-depth explanation of RADSEG, emphasizing its integration into the Barcelona Clinic Liver Cancer (BCLC) paradigm and comparing its safety and efficacy to conventional locoregional cancer treatments, with an eye on future directions and considerations.

## 2. Technical Considerations

**Figure 1:**
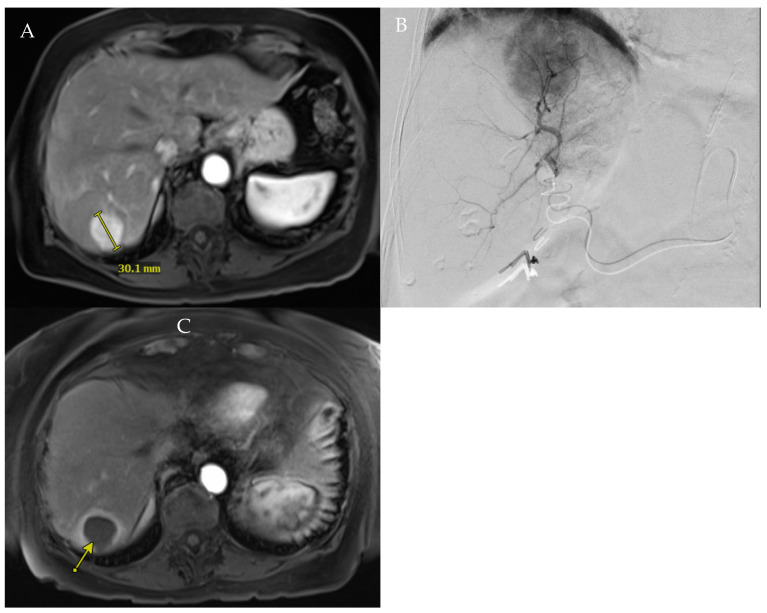
(**A**). Baseline MRI showing hyper-enhanced 3 cm tumor in segment 7. (**B**). Angiography of the segment 7 branch of the posterior right hepatic artery confirmed tumor perfusion. (**C**). Patient underwent RADSEG, with 1-month follow-up showing complete response of treated tumor (arrow).

RADSEG is formulated to selectively deliver high radiation doses to target tumor(s) and adjacent hepatic parenchyma to a maximum of two hepatic segments, as categorized by Couinaud [2,3] (Figure 1). Hepatic parenchyma outside of these targeted segment(s) is spared, minimizing radiation injury to non-cancerous hepatic parenchyma and allowing post-treatment regeneration of the non-targeted hepatic segments [4]. Garza-Ramos et al. demonstrated no definitive percentage of liver-treated safety threshold in treating HCC patients with Albumin–Bilirubin (ALBI) grade 1 and Child–Pugh (CP) class A liver function, while a safety threshold of targeting no more than 14.5% of the liver volume when treating patients with ALBI grade 2 and CP class B [5].

Patient eligibility for RADSEG is determined through a comprehensive multidisciplinary evaluation involving hepatologists, medical and radiation oncologists, liver surgeons, and interventional radiologists. RADSEG is used to bridge or downstage patients to liver transplants, and it is now gaining accepted application as a definitive therapy. Patient preparation typically includes planning angiography with lung shunt calculation following technetium-99m-macroaggregated albumin administration. However, the same-day radioembolization paradigm can be considered, given the low risk of radiation pneumonitis secondary to non-elevated lung shunt fractions along with limited hepatic volume targeted [6].

Emerging techniques are being developed to optimize RADSEG by minimizing the exposure of nontarget liver parenchyma, especially in patients with limited hepatic reserve. These techniques potentially allow for a more selective RADSEG by truncating the native liver parenchyma perfused via the intended treatment vessel distal to the tumor (flow diversion) and reducing normal liver exposure with nonselective radiation. Core et al. demonstrated the safety and practicability of this technique, reducing the exposure of nontarget hepatic parenchyma [7].

## 3. Radiation Segmentectomy Dosimetry and Rad-Path Correlation

Yttrium-90 exerts its effects primarily by undergoing β-decay to stable zirconium-90, which releases high-energy β-particles [8]. The average energy released is 0.9267 mega electron volts (MeV), with an average penetration into surrounding tissues of 2.5 mm [8]. The half-life of Y90 is 64.04 h, which means that roughly 94% of the Y90 radiation is delivered within 11 days of treatment [8]. 

Absorbed dose is defined as the amount of energy (J) deposited within a specific amount of mass (kg) and is measured as gray (Gy). It is dependent on four factors: (1) activity is measured as radioactive decay per unit time, decays per second, or Becquerel (Bq), (2) the volume in which the activity is contained, (3) distribution, with heterogenous patterns due to variations in vascular compartments and tumor angiogenesis, and (4) the radiation susceptibility of both the tumor and the hepatic parenchyma. 

### 3.1. The Medical Internal Radiation Dose (MIRD) Model

The single-compartment MIRD model is frequently used for glass microspheres for RADSEG and utilizes the assumption of a uniform distribution within the targeted tissue; therefore, only the decay in activity that occurs because of isotope decay is considered. This may result in large tumors being partially treated, or diseased liver and lung deviating significantly from the ideal density.

$$A_{0}\left[GBq\right]=\frac{D\left[\mathrm{Gy}\right]\times m[\mathrm{kg}]}{50\times (1-\frac{LSF\;\left[\%\right]}{100\;\left[\%\right]})}$$

In the equation above, “*m*” represents the mass of the tissue absorbing the radiation. *A* is the source of activity, with $A_{0}$ signifying the isotope activity at the time of calibration. “*D*” denotes the absorbed dose. Lung shunt fraction (*LSF*) correction is implemented into the formula.

### 3.2. The Partition Model

Created by Ho et al. in 1996, the partition model is a three-compartment model that considers the tumor, non-tumor, and lung tissue compartments into the MIRD equation [9]. This precise and complex model can be used for both glass and resin microspheres. It addresses preferential distribution of radiation to tumor, normal liver, and lung compartments. This model enables the prevention of liver toxicity by separating total activity into tumor ($A_{T})$ and nontumor ${(A}_{N}$) tissue. Dosimetry data can be effectively visualized using dose volume histograms to aid in adjustments for liver and tumor. However, it presents technical challenges and relies on technetium-99m macroaggregated albumin (Tc-MAA) as a surrogate for microsphere distribution in the tumor. Additional limitations include the assumption that there will be a homogenous Y90 distribution with the respective tumor and nontumor tissue compartment.

$$A_{Total}\left[GBq\right]=\frac{D_{N}\left[\mathrm{Gy}\right]\{(R_{\frac{T}{N}}{\times m_{T\left[\mathrm{kg}\right]})\;m}_{N}\left[\mathrm{kg}\right]\}}{50\times (1-\frac{LSF\;\left[\%\right]}{100\;\left[\%\right]})}$$

Here, ($R_{\frac{T}{N}}$) represents absorbed doses in the tumor ${(D}_{T})$ and nontumor ($D_{N})$ compartments. *LSF* correction is implemented into the formula. 

### 3.3. Ablative Dosimetry for RADSEG

Curative-intent therapy with RADSEG delivers an ablation dose to a targeted area encompassing a maximum of 2 adjacent hepatic segments. Most studies evaluating its efficacy have mainly used glass microspheres. Given its precision in targeting a smaller area, the assumption is that the Y90 microspheres will achieve a uniform ablation through a homogenous distribution, making the MIRD model the predominant choice. However, the partition model may be indicated when Tc-MAA imaging reveals a non-uniform Y90 distribution or when the $R_{\frac{T}{N}}$ exceeds two; this suggests that an ablative dose to the tumor ${(D}_{T})$ can be achieved with a reduced normal tissue dose $\left(D_{N}\right)$. A higher $R_{\frac{T}{N}}$ indicates a more favorable therapeutic outcome, as it signifies a greater dose concentration in the tumor relative to the surrounding normal tissues. To enhance the $R_{\frac{T}{N}}$ ratio, strategies for reducing the normal tissue dose become paramount. One effective approach is the adjustment of variables, such as the activity (GigaBq), at delivery. Fine-tuning parameters, like activity at delivery, allow for a more precise modulation of the dose distribution, potentially achieving a higher $R_{\frac{T}{N}}$ ratio by minimizing the impact on normal tissues.

The dose threshold needed to create this ablative effect has been increasing over the years as newer literature has shown increased efficacy with higher doses, while maintaining an acceptable safety profile. This is supported by explant studies assessing the levels of tumor explant complete pathologic necrosis (CPN) in patients treated with RADSEG prior to liver transplantation or surgical resection. The current dose threshold is >400 Gy, as depicted in the formula below.

$$A\left[GBq\right]=\frac{>400\;\mathrm{Gy}\times m}{50}$$

### 3.4. Complete Pathologic Necrosis (CPN)

In 2014, Vouche et al. conducted one of the first trials describing RADSEG, targeting treatment-naïve, solitary HCC ≤ 5 cm that was not amenable to resection or ablation [10]. This study aimed to evaluate the relationship between the dose administered and various outcomes, including the response rate, time-to-progression as determined by the modified Response Evaluation Criteria in Solid Tumors [mRECIST], and the connection between imaging and pathologic findings upon explant, along with the overall long term survival of the patients [10]. Among the 102 patients, 47% achieved a complete response, 39% showed a partial response, and 12% maintained stable disease. Upon explant, pathology revealed 100% necrosis, also referred to as complete pathologic necrosis (CPN), in 52% of patients, with necrosis levels ranging from 50 to 99% in the remaining 48% of patients. These outcomes provided early evidence of the association between the radiological response and CPN, demonstrating the effectiveness of RADSEG to effectively target tumor cells. Radiation absorbed doses to the volume of perfusion exceeding 190–200 Gy were associated with improved survival benefit and local tumor control. 

In 2021, Gabr et al. delved deeper into the correlation between the absorbed dose and CPN in solitary HCC ≤ 8 cm [11]. This multicenter analysis evaluated 45 explants in patients who underwent RADSEG between 2014 and 2017, and who subsequently received liver transplantation (76%) or surgical resection (24%) [11]. The median absorbed radiation dose in this study was 240 Gy, which resulted in CPN in 67% of cases, extensive necrosis in 22%, and partial necrosis in 5% of the explanted tumors. Of the patients who received a dose greater than 190 Gy, 86% exhibited CPN on explant. This is in comparison to only 65% CPN for those who received a dose of less than 190 Gy. Additionally, all patients who were administered a dose of greater than 400 Gy achieved CPN, suggesting that doses exceeding 400 Gy may represent the new threshold for consistently achieving CPN. 

In 2022, Montazeri et al. sought to evaluate whether intensification of the absorbed dose of 90Y glass microsphere RADSEG was associated with increased rates of CPN upon the explant of 75 tumors [12]. Using the MIRD dosimetry model, a dose of at least 400 Gy was used. A microsphere specific activity of ≥297 Bq was administered to the intensification group when possible. The median dose and specific activity for the treatment intensification group was 536 Gy and 715 Bq, respectively, with CPN achieved in 49% of explanted tumors. This is in contrast to the baseline cohort who received a median dose of 314 Gy and specific activity of 321 Bq, with a CPN rate of 49%. This study expanded the boundaries of threshold dose ≥ 446 Gy as there was a statistical difference between dose and CPN. Additionally, a specific activity ≥ 327 Bq was the most statistically significant independent treatment parameter associated with CPN [12]. The concept behind the use of a higher administered activity is that it increases the likelihood of decay events, thereby generating higher-energy β-particle events that can penetrate deeper into the tumor, thereby enhancing the distribution of sublethal doses. 

In 2020, DiNorcia et al. characterized predicters of explant CPN for patients undergoing locoregional therapies (LRTs) [13]. This large-scale study evaluated 3439 patients, with 32% of them achieving CPN. This study aimed to determine whether the extent of tumor response to LRTs could serve as a predictive factor for the success of liver transplantation and patient outcomes post-transplantation. Their findings elucidated a strong association between the degree of pathological response to LRT and positive patient outcomes after liver transplantation for HCC. Notably, CPN was significantly associated with lower post-transplant recurrence and superior survival. Additionally, compared to patients without CPN, patients with CPN were younger, had lower MELD and AFP levels, and were found to have tumors within Milan criteria and fewer treatments with LRT [13]. 

## 4. Radiation Segmentectomy Current Guidelines and Patient Selection

The Barcelona Clinic Liver Cancer (BCLC) is the most commonly used framework for categorizing, prognosticating, and providing treatment recommendations for HCC [14,15,16,17,18,19,20]. Stages include very early (BCLC-0), early stage (BCLC-A), intermediate stage (BCLD-B), advanced stage (BCLC-C), and end stage (BCLC-D) [21]. Factors considered include (1) tumor characteristics, including size, number, vascular invasion, and extrahepatic disease; (2) performative status as defined by the Eastern Cooperative Oncology Group (ECOG) scale; and (3) liver function with a Child–Pugh score calculated by using the lab values for bilirubin, albumin, INR, and the presence of ascites [22]. Therapies outlined in the guidelines include liver transplantation, surgical resection, thermal ablation, systemic therapies, and intra-arterial therapies with transarterial chemoembolization (TACE) or TARE. 

Very early-stage HCC (BCLC-0) is defined as a solitary lesion ≤ 2 cm without vascular invasion or extrahepatic spread in patients with preserved liver function and no cancer-related symptoms [21]. Surgical resection is preferred in certain regions due to comparable survival to liver transplantation and availability of organs, despite high recurrence rates [21]. For patients suffering from advanced liver decompensation and dysfunction, liver transplantation is the optimal treatment due to a higher priority status on the waitlist. Thermal ablation with microwave ablation (MWA) or radiofrequency ablation (RFA) would be considered first line therapy as it has comparable outcomes to those of surgical resection [21]. 

Early stage HCC (BCLC-A) encompasses solitary HCC, regardless of size, or up to three lesions measuring up to 3 cm, with preserved liver function, and without extrahepatic spread or cancer-related symptoms. Treatment of solitary BCLC-A HCC is largely based on the presence or absence of clinically significant portal hypertension (CSPH) due to its association with higher rates of postoperative complications [14,21]. Surgical resection is the preferred choice for those without CSPH. Thermal ablation is recommended for HCC up to 3 cm in an amenable location. 

Thermal ablation: Thermal ablative techniques, such as RFA and MWA, have demonstrated effectiveness for lesions up to 3 cm [23]. The goal is to achieve thorough coagulation of the tumor to ensure eradication of all malignant cells with sufficient margins. Geographic limitations may pose challenges and can lead to incomplete necrosis as well as higher recurrence rates [23]. 

TACE is a standard locoregional therapy for intermediate-stage HCC but has now been incorporated with treatment-stage migration to the early stage when resection and ablation are not options. It includes conventional TACE (c-TACE) and drug-eluting beads TACE (DEB-TACE). However, TACE may fail or become refractory [24,25]. Combining TACE with thermal ablation (TACE-ablation) has improved OS; however, repeated TACE treatments can compromise liver function [26]. 

TARE: As per the BCLC guidelines, if surgical resection and ablation are not feasible, RADSEG can be considered in solitary HCC ≤ 8 cm. For individuals on the transplant list who anticipate a waiting period of over 6 months, TARE has shown to be an effective bridge therapy with the intent to increase the number of patients making it to transplant, with prolonged time to second treatment and targeted tumor progression free survival (PFS) compared with conventional treatments. Additionally, patients with solitary HCC up to 8 cm can receive TARE with a curative intent for those not eligible for resection or ablation, or as an alternate therapy. While TARE was only recently incorporated into the BCLC guidelines in 2022, multiple studies through the years continue to demonstrate the efficacy of radiation segmentectomy as a curative treatment option [27,28,29]. Recommendations for treatment with RADSEG are made with consideration of performance status, treatment intent, tumor biology and stage, as well as hepatic reserve. In summary, the ideal case for RADSEG treatment is a solitary HCC tumor up to 8 cm in diameter unamenable to resection or thermal ablation with Child–Pugh score A cirrhosis, well-compensated liver function, no macrovascular invasion, and good performance status. 

## 5. RADSEG vs. Transarterial Chemoembolization (TACE)

Transarterial chemoembolization (TACE) is currently the most widely used primary treatment of unresectable HCC [30]. The role of TACE was confirmed by two randomized controlled trials showing therapeutic efficacy [31,32]. A meta-analysis of six randomized controlled trials demonstrated improved survival compared with best supportive care or suboptimal therapies [33]. As per the BCLC 2022 classification, TACE is a first-line treatment for patients with intermediate-stage HCC and is an option for early stage HCC patients when the initial recommended treatments have failed or are impractical [21]. Similarly, the European Association for the Study of the Liver (EASL) guidelines provides many recommendations with a high level of evidence of the use of TACE for patients with intermediate-stage HCC [34]. 

Several contemporary studies have demonstrated favorable results of RADSEG compared with TACE for treating HCC. Premiere, a prospective phase 2 randomized controlled trial compared TARE to conventional TACE (cTACE) with Doxorubicin in Lipiodol for treating BCLC stages A/B unresectable HCC unsuitable for thermal ablation [35]. Conducted between October 2009 and October 2015, this study involved 43 patients, 24 receiving TARE (selective treatment in 17 patients) and 19 undergoing TACE (selective treatment in 16 patients). It demonstrated significantly increased time to progression (TTP) of the TARE group (>26 months) compared to the TACE group (6.8 months) (*p* = 0.0012), potentially decreasing the likelihood of patients being removed from transplant waiting lists. This trial also reported adverse effects such as diarrhea (21% in the TACE group vs. 0% in the TARE group; *p* = 0.031) and hypoalbuminemia (58% in the TACE group vs. 4% in the TARE group; *p* ≤ 0.001), with the TARE group exhibiting a more favorable profile [35]. In a single-center retrospective propensity score-matched study from 2010 to 2015, Padia et al. compared RADSEG and segmental TACE for treating localized, unresectable HCC unsuitable for ablation. This study included 101 patients with 132 tumors undergoing RADSEG and 77 patients with 103 tumors receiving segmental TACE. Notably, RADSEG was associated with higher complete response rates (92% for index and 84% for overall response) compared to segmental TACE (74% and 58%, respectively) (*p* = 0.001 and *p* < 0.001) and lower tumor progression rates at 1 and 2 years. Furthermore, the median PFS was significantly longer in the RADSEG group (564 days) versus the chemoembolization group (271 days) (*p* = 0.002) [36]. Subsequently, Biederman et al. included 534 patients treated with radioembolization and 877 patients treated with TACE, and showed an improved imaging response (81.2% for TARE group vs. 49.1% for TACE group; *p* < 0.001) and median time to second treatment (700 days for TARE group vs. 246 days for TACE group; *p* = 0.009) in patients treated with RADSEG compared to those treated with TACE in the management of unresectable, solitary HCC ≤ 3 cm [37]. Most recently, the TRACE study, a single-center prospective randomized controlled trial, compared TARE to TACE for treating intermediate-stage HCC and early stage unresectable HCC that was not amenable to thermal radiation. Conducted from September 2011 to March 2018, it involved 38 participants for TARE and 34 for TACE. TARE demonstrated superior efficacy with a median TTP of 17.1 months versus 9.5 months for TACE (*p* = 0.002) and a median overall survival (OS) of 30.2 months compared to 15.6 months in the TACE group (*p* = 0.006) [38] (Table 1).

## 6. RADSEG vs. Thermal Ablation

According to the BCLC 2022 classification, percutaneous thermal ablation is considered the first-line treatment option for very early stage and early stage HCC [42]. A multicenter randomized controlled trial evaluating the efficacy of radiofrequency ablation versus surgical resection for small HCC (≤3 cm) revealed both methods to be safe therapeutic approaches with similar recurrence-free survival (RFS) rates [43]. Likewise, a second randomized clinical trial demonstrated no superiority of hepatic resection over radiofrequency ablation regarding tumor recurrence, OS, and disease-free survival for early stage HCC [44]. 

Studies have compared the effectiveness of RADSEG with percutaneous thermal ablation in treating solitary HCC. Biederman et al. conducted a retrospective study comparing the outcomes of RADSEG and TACE combined with MWA in treating unresectable solitary HCC up to 3 cm. This study, conducted from January 2010 to June 2015, involved 417 patients undergoing RADSEG and 235 undergoing TACE MWA [39]. A cohort of 121 patients (RADSEG, 41; TACE MWA, 80) with solitary HCC up to 3 cm, who had not previously undergone local-regional therapy, was identified. Propensity score matching accounted for pretreatment clinical, laboratory, and imaging covariates. In the matched cohort, the overall complete response rate, TTP, and OS were not significantly different between the two groups, with a 90-day postoperative mortality rate of 0% in both. This study concluded that imaging response and progression outcomes for patients with solitary HCC up to 3 cm treated with RADSEG were not significantly different compared to those treated with TACE MWA [39]. A second retrospective propensity score-matched study conducted from 2014 to 2017 on 68 consecutive treatment-naïve patients with solitary HCC ≤ 4 cm compared the efficacy and safety of RADSEG and percutaneous microwave ablation (MWA). No significant differences were observed in toxicity, objective tumor response, OS, and non-target liver PFS between the two groups [40]. In the matched cohort, the objective tumor response was slightly higher in the RADSEG group (90.9%) compared to the MWA group (82.6%), but this difference was not statistically significant (*p* = 0.548). The mean OS was 44.3 months for MWA and 59.0 months for RADSEG (*p* = 0.203). Notably, the targeted tumor mean PFS for the MWA group was 38.6 months, which is significantly shorter than the 57.8 months observed in the RADSEG group (*p* = 0.005). This study concluded that RADSEG achieves a similar tumor response and OS with comparable safety to MWA in managing HCC lesions ≤ 4 cm, with an added advantage of prolonged targeted tumor PFS in the RADSEG group [40] (Table 1). 

## 7. Radiation Segmentectomy Versus Surgical Resection

For BCLC 0-A HCC, liver transplantation and surgical resection have long shown to be effective therapies for this patient population, with 60–80% 5-year survival rates [19]. While the efficacy of surgical resection has long been established, there are inherent limitations. The 5-year recurrence rate after resection can range anywhere from 40 to 70% [45,46,47]. Additionally, surgery is only feasible in patients who meet strict eligibility criteria and lack complicated medical comorbidities. This is particularly true as hepatectomy poses excessive risks in patients with cirrhosis, which is commonly seen in patients with HCC. These associated risks, including decompensated liver failure and mortality, can further jeopardize the patient’s eligibility for liver transplantation, should it become necessary. All the while, RADSEG has emerged as a potential curative alternative, especially in patients who may not be immediate candidates for surgery. Although the direct comparative studies between surgical resection and RADSEG are limited, it is essential to explore the recent literature that has elucidated the curative potential of RADSEG. 

A study conducted in 2014 laid the initial groundwork, highlighting the association between RADSEG and CPN in HCC with a dose of > 190 Gy [10]. Building upon this, Lewandowski et al. in 2018 retrospectively evaluated patients with solitary HCC ≤ 5 cm who underwent RADSEG with a dose of >190 Gy in lesions in cases where surgical resection was not a viable option [27]. This study was one of the first to note findings in line with other curative therapies, such as surgical resection. Using the guidelines set by the European Association for the Study of the Liver (EASL), 90% of patients were responsive, with 59% showing a complete response. Local tumor control rate at 5 years was 72% with a median time to progression of 2.4 years. The overall 5-year survival rate was reported at 55%, and up to 75% for lesions ≤ 3 cm, which is in accordance with the data seen with other curative-intent treatments [27]. 

In 2021, the LEGACY study evaluated objective response rates and duration of responses in 162 patients who underwent TARE in the treatment of solitary, unresectable HCC up to 8 cm [28]. In patients who received neoadjuvant TARE, 21% were prior to transplantation and 6.8% were prior to resection, with the remaining undergoing the primary treatment. The majority of patients had BCLC-A HCC (60.5%) and 39.5% of them had BCLC-C HCC. Most patients (95.7%) received selective infusions; however, 1.9% received lobar and 2.5% received mixed infusions. The median tumor size was 2.7 cm. Using mRECIST criteria, the best objective response rate was 88.3%, with confirmed objective response rates up to 72.2%. Moreover, 76.1% of participants maintained their duration of response for 6 months or longer. For patients who did not receive liver transplantation or resection, their overall survival mirrored that of surgical resection, with nearly 70% still alive at the 60-month mark post-treatment [19,28]. The favorable outcomes seen with this study were instrumental in the integration of TARE into the BCLC guidelines. 

In 2022, the RASER trial was the first prospective trial to explicitly evaluate the efficacy and safety of RADSEG in the treatment of very early to early stage HCC (BCLC 0-A) [29]. In total, 29 patients with solitary HCC ≤ 3 cm, all of whom deemed to have surgically unresectable disease, received RADSEG [29]. The trial reported an initial target lesion complete response of 83% of patients, with 90% experiencing a sustained complete response after a single treatment. Further demonstrating the curative potential of radiation segmentectomy, 8 explants at time of transplantation exhibited 100% CPN after just one treatment. This is likely attributed to the high radiation doses delivered to the tumors, which were seen to be more than 1000 Gy on post-PET/CT. Moreover, the trial displayed a favorable safety profile with only two non-laboratory Clavien-Dindo 3 adverse events. These outcomes align with the findings from prior retrospective studies evaluating RADSEG in BCLC-0-A HCC [10,27,28]. 

A multisite retrospective analysis conducted by De la Garza-Ramos et al. in 2022 was one of the first to directly compare RADSEG to surgical resection [41]. It involved 123 treatment-naïve patients with HCC ≤ 8 cm, ECOG 0-1, with absence of macrovascular invasion or hepatic disease. Of these patients, 57 received radiation segmentectomy, while 66 underwent surgical resection. This study showed tumor progression in 5% of patients who underwent RADSEG versus 7% in the surgical resection group [41]. There was a significant difference in time to progression of 21.9 months for RADSEG and 29.4 months for those who received surgical resection. It is important to note there were significant disparities between the two cohorts in terms of tumor size, Child–Pugh class, Albumin–Bilirubin score, platelet count, and fibrosis stage. When accounting for differences, subgroup analyses showed no significant differences in time to progression between the two treatment groups for cohorts with fibrosis stages 3–4 and a platelet count below 150. Additionally, in patients who were ineligible for hepatectomy, the RADSEG group exhibited a lower incidence of major adverse events, measured as Clavien-Dindo 3 and above. 

The goal of RADSEG, like surgical resection, is to cure the patient, and it has demonstrated feasibility through higher dose thresholds resulting in ablative therapy with resultant CPN on explant, as seen repeatedly in the literature in recent years [45,46,47]. At this moment there is insufficient data to consider radiation segmentectomy on par with surgical resection. Surgical resection remains the first-line therapy in patients with early tumors, well-preserved liver function, and no clinically significant portal hypertension, with RADSEG serving as a promising alternative for patients ineligible for surgery. Moving forward, more studies are needed to directly compare radiation segmentectomy to surgical resection, with an emphasis on dose optimization, efficacy, and survival. 

Nevertheless, RADSEG remains a promising treatment in BCLC 0-A HCC, particularly for patients for whom surgical options are not viable. With the capacity to deliver high doses of radiation directly to the tumor, while sparing healthy liver tissue, RADSEG offers both a curative and safe alternative. Studies such as LEGACY and RASER have suggested that RADSEG can even achieve outcomes comparable to surgical resection, likely due to its ability to reach high rates of complete pathologic necrosis [11,12,13]. The study conducted by De la Garza-Ramos et al., which directly compares RADSEG and surgical resection, further substantiates this efficacy. As the body of research continues to expand, the goal is for the BCLC treatment guidelines to be updated to reflect the curative role of RADSEG in the treatment of very early to early HCC (Table 1). 

## 8. Future Directions

The potential for the curative intent of RADSEG requires us to evaluate the maximum tolerated dose for this ablative therapy and more prospective study. Most of the current literature supporting RADSEG uses glass microspheres in patients with HCC. Thus, more research is required for resin microspheres and for the treatment of secondary hepatic malignancies. 

## 9. Conclusions

TARE is an evolving, expanding image-guided therapy. RADSEG is one such advancement, providing ablative radiation dosimetry to small hepatic volumes. RADSEG intends to bridge patients to liver transplantation by delaying disease progression, downstage disease to meet transplantation criteria, treat patients with macrovascular invasion, and be a definitive therapy in eligible patients with located tumors. It is defined by high local imaging response rates, durable responses with a long time to treated tumor progression, and significant rates of explant necrosis. However, as its application continues to expand, future studies are required to define the threshold dosimetry to align outcomes with other curative-intent treatments.

## Figures and Tables

**Table 1:** Summary of the comparative outcomes of RADSEG with traditional locoregional treatments for HCC.

Study	Comparison	Outcome Measures	Key Findings
Salem et al. (2016) (Premiere Study) [35]	TARE (24 patients) vs. cTACE (19 patients) with Doxorubicin	TTP, safety	TARE group demonstrated significantly increased TTP (>26 months) vs. cTACE (6.8 months) (*p* = 0.0012), fewer adverse effects such as diarrhea (*p* = 0.031), and hypoalbuminemia (*p* < 0.001) in the TARE group
Padia et al. (2017) [36]	RADSEG (101 patients) vs. segmental TACE (77 patients)	Complete response rates, tumor progression rates, PFS	RADSEG associated with higher complete response rates (92% index (*p* = *0*.001), 84% overall (*p* < 0.001)), lower tumor progression rates at 1 and 2 years, and significantly longer median PFS (564 days vs. 271 days) (*p* = 0.002)
Biederman et al. (2018) [37]	RADSEG (534 patients) vs. TACE (877 patients)	Imaging Response, Time to Second Treatment	Improved imaging response (81.2% vs. 49.1%) (*p* < 0.001) and median time to second treatment (700 days vs. 246 days) (*p* = 0.009) in the RADSEG group
Dhondt et al. (2022) (TRACE Study) [38]	TARE (38 patients) vs. TACE (34 patients)	TTP, OS	TARE demonstrated superior efficacy with a median TTP of 17.1 months vs. 9.5 months for TACE (*p* = 0.002) and a median OS of 30.2 months vs. 15.6 months in the TACE group (*p* = 0.006)
Biederman et al. (2017) [39]	RADSEG (417 patients) vs. TACE combined with MWA (235 patients)	Complete response rate, TTP, OS	No significant differences observed in complete response rate (*p* = 0.94), TTP (*p* = 0.83), or OS (*p* > 0.99) between RADSEG and TACE combined with MWA groups
Arndt et al. (2021) [40]	RADSEG (34 patients) vs. MWA (34 patients)	Objective tumor response, OS, targeted tumor PFS	RADSEG achieved similar objective tumor response (*p* = 0.548) and OS (*p* = 0.203) with comparable safety to MWA, with prolonged targeted tumor PFS (*p* = 0.005)
De la Garza-Ramos et al. (2022) [41]	RADSEG (57 patients) vs. surgical resection (66 patients)	Target tumor and overall progression, TTP, OS	No significant difference in overall progression (*p* = 0.71) and TTP (*p* = 0.29) between RADSEG and surgical resection groups after adjusting for covariates; the median OS was not reached for either of the groups
